# Single shot quantitative polarized light imaging system for rapid planar biaxial testing of soft tissues

**DOI:** 10.3389/fbioe.2022.1010307

**Published:** 2022-09-21

**Authors:** Michael J. Blair, Kyle P. Quinn

**Affiliations:** Department of Biomedical Engineering, University of Arkansas, Fayetteville, AR, United States

**Keywords:** polarized light, skin, collagen, birefringence, biomechanics, polarimetry

## Abstract

Quantitative Polarized Light Imaging (QPLI) is an established technique used to compute the orientation of collagen fibers based on their birefringence. QPLI systems typically require rotating linear polarizers to obtain sufficient data to estimate orientation, which limits acquisition speeds and is not ideal for its application to mechanical testing. In this paper, we present a QPLI system designed with no moving parts; a single shot technique which is ideal to characterize collagen fiber orientation and kinematics during mechanical testing. Our single shot QPLI system (ssQPLI) sorts polarized light into four linear polarization states that are collected simultaneously by four cameras. The ssQPLI system was validated using samples with known orientation and retardation, and we demonstrate its use with planar biaxial testing of mouse skin. The ssQPLI system was accurate with a mean orientation error of 1.35° ± 1.58°. Skin samples were tested with multiple loading protocols and in each case the mean orientation of the collagen network reoriented to align in the direction of primary loading as expected. In summary, the ssQPLI system is effective at quantifying collagen fiber organization, and, when combined with mechanical testing, can rapidly provide pixel-wise measures of fiber orientation during biaxial loading.

## Introduction

The structure and function of soft tissues depends considerably on collagen fiber organization. Fiber orientation, directional variance, and kinematics are used to describe how the collagen fiber network reacts to mechanical loads. Previous work to understand the dynamic behavior of collagen fiber networks has shown that fibers typically reorient when loads are applied to soft tissue, and fibers align toward the direction of maximum principal strain (Lanir, 1979, Lanir, 1976; Markenscoff and Yannas, 1979). Detailed analysis of the collagen fiber orientation and kinematics in engineered tissue has been used to develop and validate multiscale fiber-network based models of mechanical behavior (Raghupathy et al., 2011; Sander et al., 2009). Collagen fiber kinematics during tensile loading have been used to detect damage in capsular ligament well before the damage was visible (Quinn and Winkelstein, 2008). Understanding the collagen fiber kinematics of tissues during mechanical loading is critical to understanding and defining structure-function relationships in biomechanics, and several imaging modalities have been adapted to do this.

Histological staining and electron microscopy were first used to describe the collagen organization of biological tissues (Bear, 1952; Holmstrand et al., 1961). However, these modalities have a distinct disadvantage in that the tissue must be fixed, sliced, and stained; as a result of these destructive processes, fiber information can only be gathered at a single time point or mechanical loading state. Several non-destructive imaging modalities have since been utilized that provide the ability to characterize the fiber structure during dynamic loading: Small angle light scattering (SALS), diffusion tensor magnetic resonance imaging (dMRI), optical coherence tomography (OCT), and nonlinear optical microscopy (Arkill et al., 2010; Hansen et al., 2000; Robitaille et al., 2011; Sacks et al., 1997; Sacks and Chuong, 1992; Wang et al., 2020; Matcher et al., 2004; Mostaco-Guidolin et al., 2017; Quinn et al., 2016; Woessner et al., 2021). However, these methods to quantify fiber orientation during testing are expensive, slow, inefficient, and/or have low spatial resolution.

Quantitative polarized light imaging (QPLI) is a relatively inexpensive and scalable imaging approach that is well suited to assess fiber population responses from entire planar tissues undergoing mechanical testing. QPLI takes advantage of the birefringent nature of collagen, which causes a phase retardation of orthogonal polarized light components transmitted through collagen fibers within tissue. QPLI is a subset of polarization techniques which use Mueller calculus to characterize tissue optical properties for various biomedical and clinical applications (Tyo et al., 2006; He et al., 2021). The speed of acquisition is increased in QPLI by assuming that collagenous tissue is a linear birefringent material. To image these materials, the typical optical train includes transmission of light through a rotating linear polarizer (LP) and a fixed circular analyzer (Tower, et al., 2002; Quinn and Winkelstein, 2008; Quinn and Winkelstein, 2009; Woessner et al., 2021). Alternatively, light can be passed through the birefringent sample and two rotating crossed-polarizers (Lake et al., 2009). Relative to imaging systems that require raster scanning, QPLI systems involving polarizers rotating over angles of at least 180° are able to provide pixel-wise maps of fiber orientation. However, they still require the collection of a series of 10–20 images as the polarizer rotates to construct orientation maps, which limits mechanical testing to quasi-static speeds. High-speed cameras have been used to overcome this obstacle, but this drastically increases the price of the system and decreases the exposure time needed to collect a sufficient signal (Quinn and Winkelstein, 2008, 2009, 2011; Wu et al., 2018).

To facilitate a broad range of mechanical testing speeds, there is a need to collect photons as efficiently as possible in QPLI systems. As an alternative to methods requiring the acquisition of a series of images, a variety of snapshot techniques are capable of measuring polarization states at a single timepoint (Tyo et al., 2006; He et al., 2021). Division of aperture (DoAp) devices duplicate an image onto one sensor, each with a unique polarization state (Pezzaniti and Chenault, 2005; He et al., 2015). This method requires that multiple lenses are carefully aligned to focus the image onto each quarter of the sensor. Division of focal plane (DoFP) sensors implement pixel-matched polarization filters to create 2 × 2 grids of fixed polarizer angles of 0°, 45°, 90°, and 135° (York et al., 2014a; York et al., 2014b). The DoAp and DoFP systems both rely on polarizers, which filter out approximately 50% of light at a given pixel, and reduce the effective resolution of the images by partitioning pixels on the camera sensor. Alternatively, division-of-amplitude (DoAmp) devices split the incoming light into different polarization states using multiple polarized beamsplitters and focus the light onto multiple sensors (Compain and Drevillon, 1998; Azzam, 2010; Beckwith and Yang, 2021). Although DoAmp devices require careful image registration, they offer good spatial resolution and light collection efficiency.

In this paper, we present a single shot QPLI (ssQPLI) system that employs a DoAmp scheme by sorting polarized light into one of four high-resolution cameras. This system employs inexpensive and widely available optical components and has no rotating elements, which allows for the collection of fiber orientation and retardation maps from a single, synchronous collection of images from four cameras. We have validated the ssQPLI system with samples of known retardation and orientation, and demonstrate its use in measuring collagen fiber kinematics during biaxial mechanical testing of skin.

## Materials and methods

### Overview of the single shot QPLI system

**FIGURE 1:**
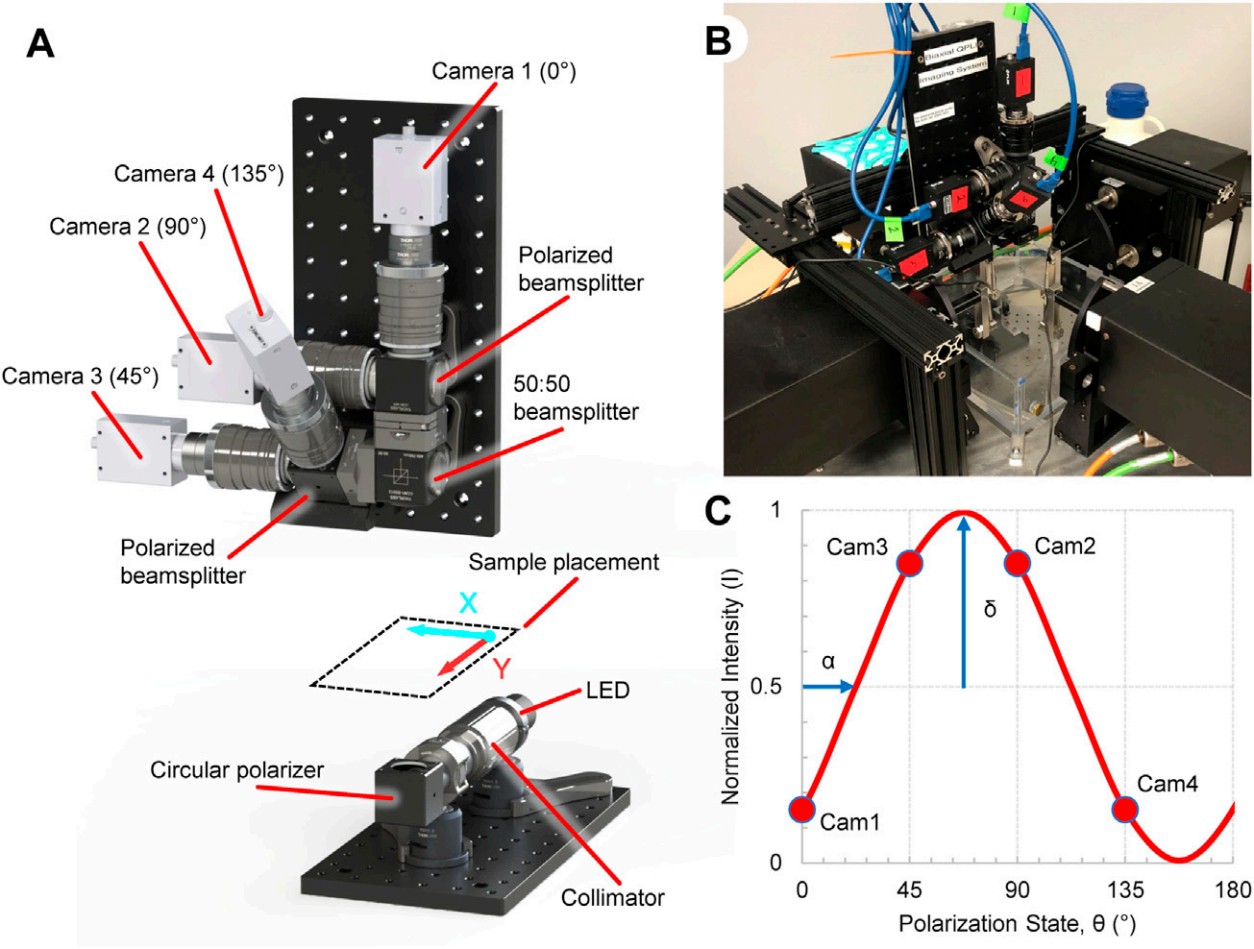
Overview of the ssQPLI system to create collagen fiber orientation maps during planar biaxial loading. **(A)** Light from a 660 nm LED is collimated and circularly polarized before being transmitted through a collagenous sample, which is sorted by polarizing beamsplitters. Four cameras simultaneously image the sorted light. **(B)** The ssQPLI system mounted to a planar biaxial testing machine with the horizontal axis of the images aligned with the XX-axis of the machine. **(C)** The intensity of light will fluctuate depending on the linear polarizer orientation, and the intensity of the four cameras with fixed beamsplitter orientations can be used to extract collagen fiber orientation (α) and retardation (*δ*).

The ssQPLI system was built around and mounted to a planar biaxial test machine (574 Series, TestResources, Shakopee, MN) to analyze the collagen alignment in planar biological samples (Figure 1B). An illumination source was assembled to emit circularly polarized light (CPL) and transmit it though a sample. Light is generated by a 660 nm LED (ThorLabs, Newton, NJ), collimated, and passed through a circular polarizer (CP, Figure 1A) consisting of a linear polarizer and a quarter wave plate (670 nm, ThorLabs) rotated at 45° relative to the linear polarizer. The CPL then passes through collagenous tissue which acts as a linear birefringent element, resulting in elliptically polarized light (Tower and Tranquillo, 2001; Blanpain et al., 2007). A collection unit gathers and sorts the elliptically polarized light from the sample. The collected light is first passed through a 670 nm bandpass filter (FWHM = 10 nm, ThorLabs). A 50:50 non-polarized beamsplitter (BS013, ThorLabs) splits the light and sends each half into one of two polarized beamsplitters (PBS251, ThorLabs). Four cameras (FLIR Grasshopper 3) with lenses (50 mm, F/1.4, ThorLabs) were attached to the outlets of each of the polarized beamsplitters. The beamsplitter/camera units were oriented such that the collected light is sorted based on linear polarization states oriented at 0°, 45°, 90°, and 135° from the horizontal axis of the cameras. The images (1920 × 1,200, 8-bit) were captured and saved using a custom C++ program. The field of view for the four cameras was 5.3 × 3.3 cm, with a resolution of 36 pixels/mm, and images were acquired at 50 fps. A dedicated four port PCIe USB card (StarTech, Groveport, OH) was used to capture the camera data. A digital signal produced by an Arduino Uno was used to trigger each of the cameras simultaneously, and was also supplied to the biaxial machine to synchronize the camera and biaxial systems. A custom Matlab program (2020b, MathWorks, Natick, MA) was developed to read image timestamps and align them with the Arduino signal pulse recorded by the mechanical testing machine.

### Theory

The orientation of collagen fibers was determined by harmonic analysis, as described previously (Glazer et al., 1996; Tower et al., 2002; Quinn and Winkelstein, 2008; Lake et al., 2009; Woessner et al., 2019). Briefly, when assuming linear birefringent samples, the pixel-wise intensity can be described as:

I(θ)=A+Bcos(2θ)+Csin(2θ)

where θ is the angle which corresponds to the orientation of the polarized beamsplitter relative to the horizontal, and A, B, and C are the Fourier coefficients. In traditional QPLI systems, *θ* refers to the angle of a rotating LP, and I (*θ*), the intensity of light at a given location, will sinusoidally oscillate as *θ* changes (Figure 1C). With three or more unique orientations (*θ*), A, B, and C are solved for using a summation approximation (Tower et al., 2002). Then the orientation (*α*) and retardation (*δ*) of the birefringent elements of a sample can be calculated as follows:

$$Orientation\;\left(\alpha \right)=\frac{1}{2}\tan^{-1}\left(\frac{-B}{C}\right)$$

$$Retardation\;\left(\delta \right)=\cos^{-1}\left(\sqrt{1-B^{2}-C^{2}}\right)$$

The retardation maps are used to scale the orientation maps by the corresponding magnitude of the retardation to give more weight to higher retardation values, which are indicative of more aligned collagen fibers. Using directional statistics, the mean fiber orientation and directional variance were also calculated within a region of interest (ROI) in MATLAB.

### Camera calibration

Images acquired from each camera simultaneously form an image set used to create fiber orientation and retardation maps, and images within a set were registered through digital cross-correlation to ensure an accurate pixel-wise comparison among all cameras. Next, we collected an image set composed of only the CPL produced by the illumination unit and calculated the 2nd, 3rd, and 4th Stokes vector parameters. We used these parameters to correct for any imperfections of the circular polarizer as described by Tower et al. (2002) in previous work. Additionally, the average light intensity collected from each of the four cameras was computed from an image of the CPL illumination and used to normalize pixel intensities from that camera in all subsequent images collected. Finally, any saturated pixels (typically less than 0.2% of all pixels) were removed from each image and their corresponding pixel locations in rest of the image set. The registration and normalization steps were performed prior to each test to confirm that pixel-wise comparisons of an image set remained accurate.

### Validation of orientation and retardation

**FIGURE 2:**
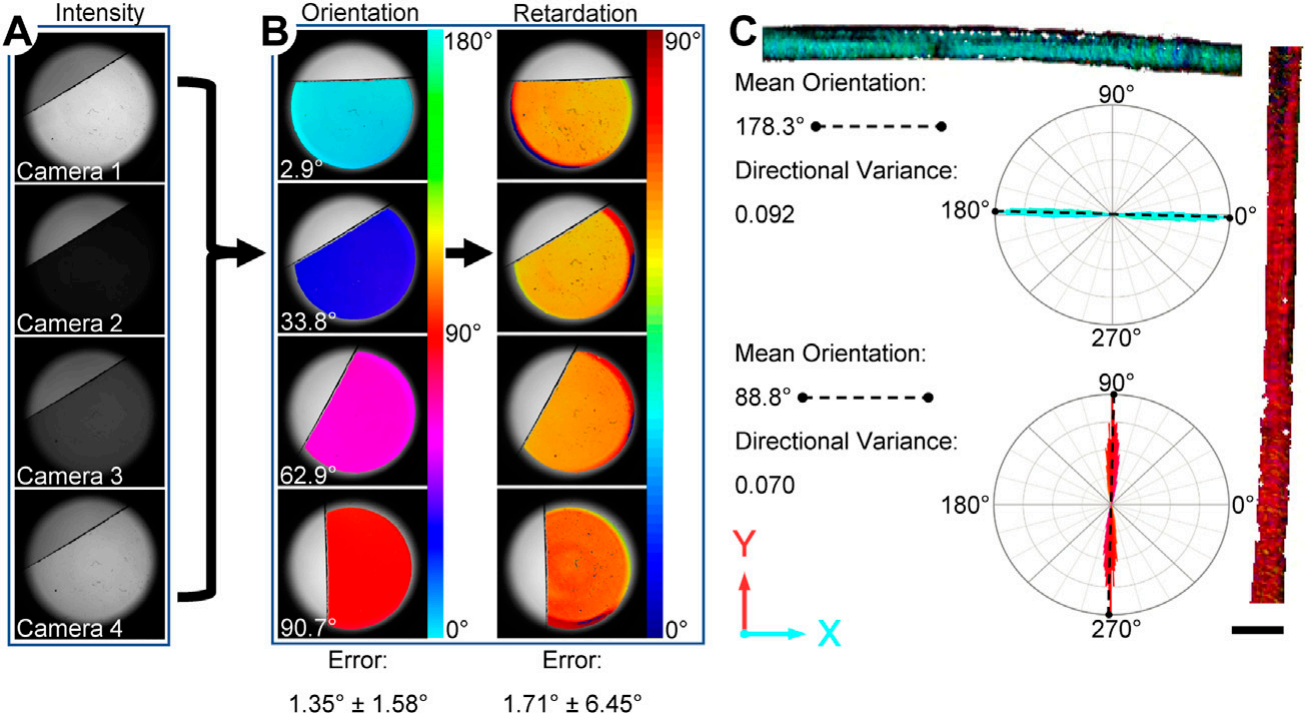
Overview of the ssQPLI measurement validation consisting of a waveplate and highly aligned rat tail collagen. **(A)** Intensity images of a waveplate from the four cameras. The intensity values were compared on a pixel-wise basis to produce orientation and retardation maps. **(B)** Four representative maps of the orientation and phase retardation of a waveplate, which was used to assess error in the ssQPLI measurements at several orientations. **(C)** Rat tail collagen was also imaged and rose plots of the orientation show that fibers were highly aligned in the expected direction. Scale bar = 1 mm.

As an initial step to assess the accuracy of the assembled system, a waveplate (140 nm, American Polarizers, Reading, PA) with a retardation of 75.2° at 670 nm, was imaged at several orientations (Figures 2A,B). Error was defined as the difference in degrees between the measured and expected values of orientation or retardation, and reported as a mean ± standard deviation. Additional validation of collagen fiber orientation was performed using highly aligned fibers extracted from a Sprague Dawley rat (male, 6 months. Old) as described previously (Bruneau et al., 2010). The collagen fibers were placed on a standard glass microscope slide and imaged with the ssQPLI system in the horizontal and vertical orientation. ImageJ was used to measure the actual orientation of the fiber bundles.

### Measuring fiber realignment during biaxial loading

C57BL/6J mouse skin was used to demonstrate the capabilities of the ssQPLI system during biaxial loading. Skin samples were collected under the University of Arkansas IACUC Protocol #17066. Full-thickness skin samples were excised from the ventral side of euthanized mice (*n* = 3, 12 weeks old, male). The samples were frozen and stored at −80°C. For testing, the samples were thawed, depilated with Nair (Church and Dwight Co., Inc., Ewing, NJ) for 5 min on the epidermal side, and trimmed of excess fat on the hypodermal side. The samples were cut into 1.6 cm squares with a custom punch, and marks were made on one edge to ensure that each sample was similarly oriented such that the rostral-caudal plane of the mouse corresponded to the horizontal plane on the ssQPLI. Prior to mechanical testing, samples were placed between a microscope slide and cover, and the thickness of the sample was measured with a dial indicator. Micro-beads were attached on the samples with cyanoacrylate glue to form a square approximately 0.7 cm per side to serve as fiduciary markers for strain tracking. The ROI for the ssQPLI analysis was defined by the position of the microbeads. The samples were kept well hydrated with phosphate buffered saline (PBS, 1X), and subsequently underwent mechanical testing.

The sample was mounted in the biaxial testing machine with four evenly-spaced hooks on each side, for a total of 16 mounting points. Once mounted, the samples were hydrated by floating on a PBS (1X) bath. A preload of 10 kPa was applied to each sample. The crosshead displacement rate was based on the camera frame rate and resolution, and was limited such that the image sets would be taken while the sample was extended no more than 0.5 pixels. The samples underwent 100 equibiaxial preconditioning cycles to 3 mm (∼15% strain) at a rate of 36 mm/min. Next, the sample was tested in a sequence of loading protocols, which are presented here as a ratio of 3 mm of total extension in the XX:YY directions: 1:1, 1:0, 0:1, 1:0.5, and 0.5:1. A total of 15 loading cycles were performed for each protocol and image sets were taken during the last loading and unloading cycle of the protocol. Load cell and displacement data were collected at 1 KHz.

The image sets from the four cameras collected at 50 fps were used to track displacement of the fiduciary markers on the sample. A custom Matlab program was used to calculate the deformation gradient tensor ( F ) from the fiduciary marker movement as previously described (Sacks, 2000). Green’s strain ( E ) was calculated as:

$$E=0.5*\left(F^{T}F-I\right)$$

where I is the identity matrix. The first Piola-Kirchhoff stress ( P ) was calculated as:

$$P=\left[\begin{array}{cc} \frac{f_{xx}}{A_{YY}} & 0\\ 0 & \frac{f_{YY}}{A_{XX}} \end{array}\right]$$

where f is the force applied in the XX or YY direction, and A is the cross-sectional area of the sample to which the force was being applied. $P_{12}$ and $P_{21}$ were assumed to be 0 because the forces were applied perpendicularly to the *X* and *Y* axes of the sample. The 2nd Piola-Kirchhoff stress (2nd PK, **S**) could then be calculated as:

$$S=F^{-1}P$$

The components $S_{11}$ and $S_{22}$ correspond to the reported XX and YY values.

## Results

The ssQPLI system demonstrated good accuracy in measuring the orientation and light retardation of a reference waveplate (140 nm). Specifically, mean orientation had an error of 1.35° ± 1.58° when compared to digitized orientation measurements in ImageJ, and mean retardation measurements had an error of 1.71° ± 6.45° when compared to product specifications (Figure 2B). The standard deviation in pixel-wise error measurements suggests potential pixel-to-pixel variability among camera sensors does not contribute to significant error in the system. Image sets collected of highly aligned collagen fibers in a rat tail sample also demonstrated the ability to create pixel-wise maps of fiber orientation in biological tissues with high accuracy. The mean value of collagen fibers oriented horizontally had an error of 0.4% compared to the mean value measured with ImageJ. The sample was subsequently rotated to a vertical orientation and the error was measured at 0.8%. The directional variance, a measure of fiber alignment with a value of 0 indicating fully aligned fibers, was 0.09 and 0.07 for the horizontal and vertical positions, respectively. The high degree of alignment of the fibers was also indicated in the narrow distribution of orientation values in the rose plots associated with the rat tail images (Figure 2C).

**FIGURE 3:**
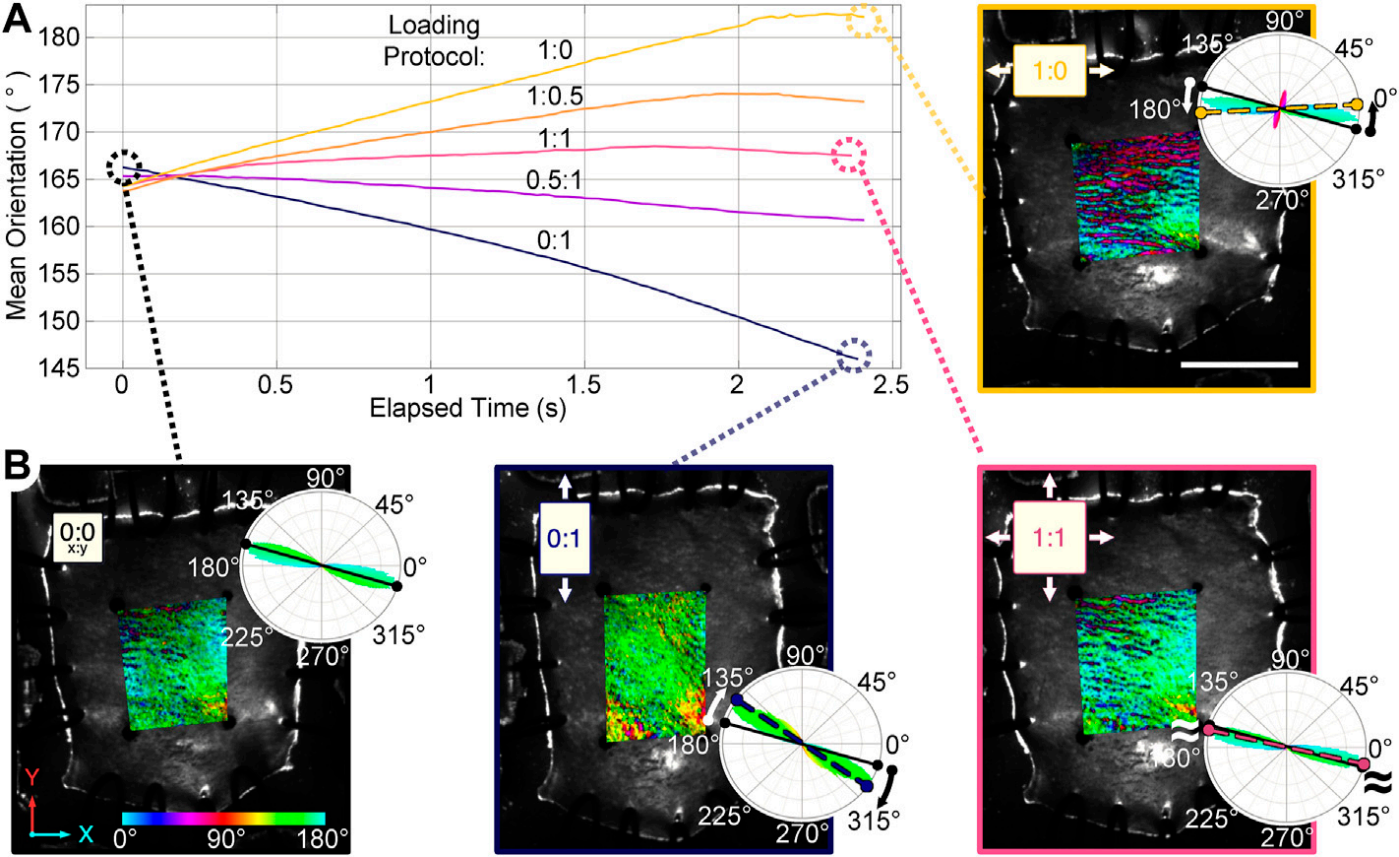
Planar biaxial testing of mouse skin samples under multiple loading protocols (XX:YY, 3 mm total travel). **(A)** The mean orientation within the central region of interest (ROI) reoriented toward the direction of greatest stretch. **(B)** Orientation maps of a sample in the undeformed configuration (0:0), and maximum strains for representative loading protocols (0:1, 1:1, and 1:0) are shown. A color bar indicates the pixel-wise direction of the fibers inside the ROI, the rose plots show fiber orientation distributions for each map. The solid line in each rose plot indicates the mean orientation of the undeformed sample, while the dashed line indicates the mean orientation at maximum strain. Scale bar = 7 mm.

Fiber orientation maps of mouse skin undergoing planar biaxial testing demonstrate an ability to measure fiber kinematics during continuous loading (Figure 3). The mean fiber orientation in the unloaded skin samples was 167° ± 9.5°. As expected, fibers reoriented toward the direction of loading when loaded in only the XX or YY directions, while equi-biaxial loading of the samples predictably exhibited little change in the mean fiber orientation. Two samples had a similar initial orientation of 161° ± 2.5°, and reoriented by similar amounts in the XX direction during the 1:0 protocol (24.3° ± 7.0°), and in the YY direction during the 0:1 protocol (22.8° ± 5.6°). The third sample was highly aligned at 178° (almost completely in the XX direction) with a low directional variance of 0.17 in the undeformed configuration. With most fibers already parallel to the direction of loading in the XX direction, they did not reorient in the 1:0 protocol. Although the mean orientation of the fiber population did not change appreciably when stretched in the YY direction during the 0:1 protocol, there was a substantial increase in the directional variance of fiber orientations from 0.17 to 0.65. This indicates the fiber population was becoming less aligned in the XX direction as it was stretched in the YY direction. Thus, despite some variability in initial fiber orientation, each sample demonstrated that collagen fiber populations responded to applied loads as one would expect by either rotating their mean direction or changing the directional variance in the mean direction.

**FIGURE 4:**
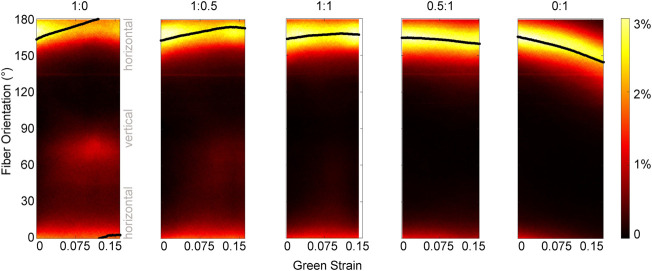
Pixel-wise fiber orientation distributions as a function of Green strain for different loading protocols. The change in the fiber orientation distribution during each loading protocol highlights different kinematics. In the 0:1 test, as the tissue is loaded in the vertical direction, the entire fiber population at 165° rotates toward the loading direction. However, fiber rotation at each pixel is not observed in the 1:0 test as the tissue is loaded in the horizontal direction. Instead, a subpopulation of fibers oriented at 165° flip their orientation to approximately 75°. Green strain is measured in the XX direction for configurations 1:0, 1:0.5, and 1:1; and in the YY direction for configurations 0.5:1 and 0:1. The scale of the color bar indicates the percent of pixels in the orientation map that correspond to a given orientation. The solid black line shows the mean fiber orientation for each map.

In addition to some sample-to-sample variability in how fiber populations responded to loading, we observed direction-dependent variability in the response within one sample. The tissue, initially with fibers primarily oriented between 150°–180° (Figure 3B), expanded in the 1:0 protocol to reveal another subpopulation of fibers oriented at approximately 70°–80° (1:0 protocol**,** magenta/red fibers). These fibers did not simply rotate toward the direction of loading as was observed in the 0:1 protocol, but were a distinct group that only appeared as force was increasingly applied in the XX direction (Figure 4). The change in fiber directional variance was generally small for the 0:1, 0.5:1, and 1:1 protocols, ranging from 0.11 to 0.25, but increased from 0.16 to 0.60 in the 1:0 protocol, in which the orthogonal fiber subpopulations appeared.

**FIGURE 5:**
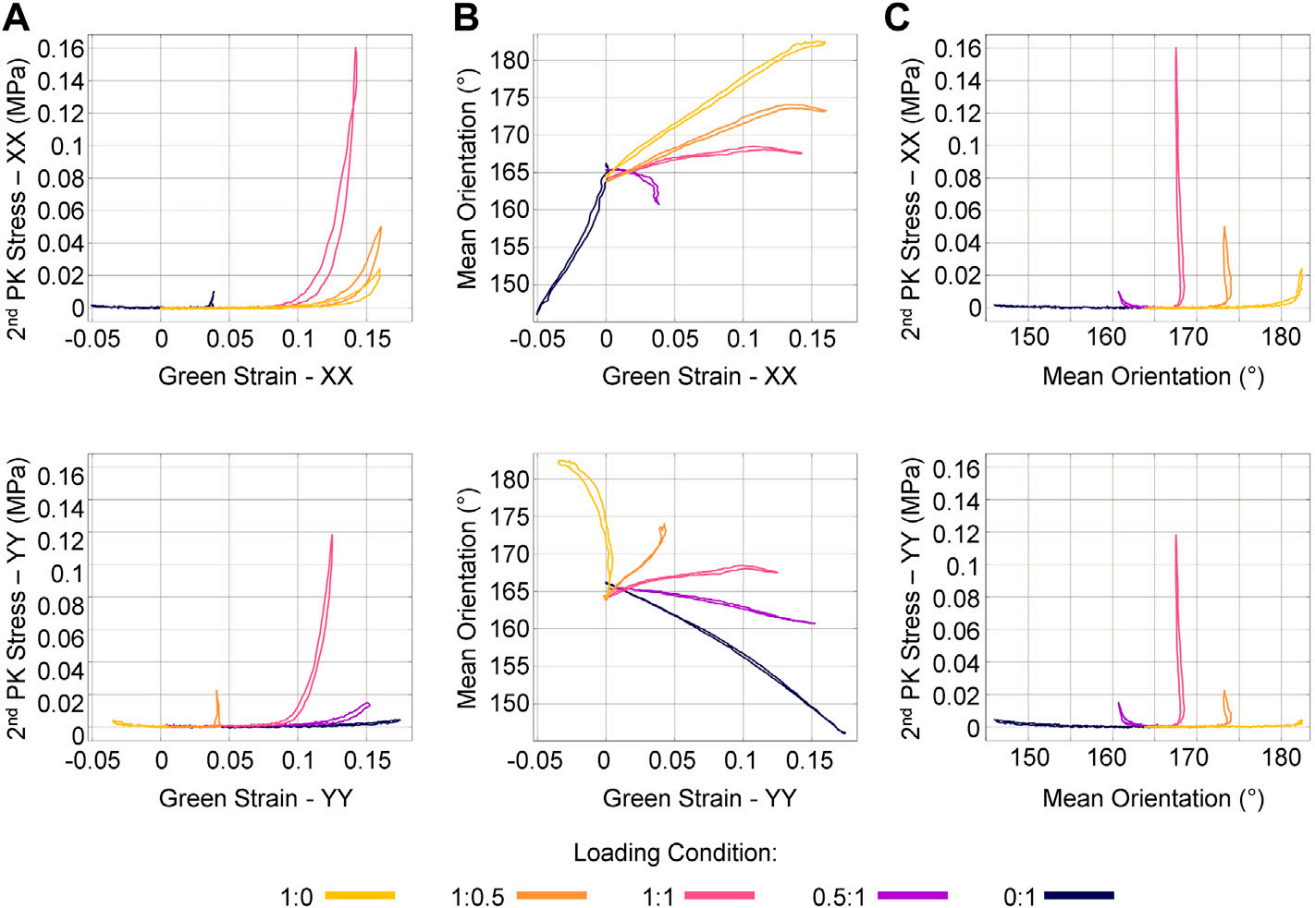
Mechanical and fiber kinematic response of a representative sample during several planar biaxial loading protocols. **(A)** Stress and strain were measured with respect to each primary loading axis (XX and YY). Notably, the stress was considerably greater in the 1:1 loading protocol. **(B)** Orientation typically changed linearly with an increase in strain, though the plateau seen in the 1:0, 1:0.5 indicates that fibers rotated as far as possible even under increasing strain. **(C)** Stress does not increase until fibers stop rotating toward the axis of loading.

By synchronizing the ssQPLI data with mechanical testing data, we were able to distinguish how fiber kinematics relate to the stress-strain response of soft tissues. The largest 2nd Piola-Kirchhoff (PK) stress occurred in the 1:1 protocol (Figure 5A), likely due to the recruitment of collagen fibers constrained by entanglement with neighboring fibers. Mean fiber orientation typically changed linearly with an increase in Green strain. Near the end of the loading cycle for the XX direction-dominated loading protocols (1:0 and 1:0.5), the orientation stopped changing with a continued increase in applied strain. This was not observed in the YY direction-dominated protocols (0:1 and 0.5:1). This behavior likely indicates that, given the loading conditions, the fibers have rotated as far as they are able in the entangled network (Figure 5B). Once fiber reorientation stopped occurring with increased stretch, stress increased significantly (Figure 5C). This behavior of collagenous soft tissues has previously been described (Lanir, 1979; Billiar and Sacks, 1997; Yang et al., 2015; Blair et al., 2020). The collagen fibers first rotate toward the axis of loading and uncrimp. Once fiber populations cannot align any further toward the direction of loading, they begin to stretch and bear significant load. The ssQPLI system was able to quickly and accurately calculate these changes in the fiber network as the mechanical test was taking place, providing detailed maps of fiber kinematic data with good temporal and spatial resolution.

## Discussion

We have developed a single shot QPLI system coupled with a planar biaxial testing machine to map the light retardation and orientation of collagen in soft tissue during continuous loading. The ssQPLI system was designed to overcome the inefficiency of previous systems. Collagen orientation information was validated with samples of known orientation, and fiber kinematics during biaxial testing were quantified and compared to the mechanical response in mouse skin. On average, collagen fibers reoriented toward the direction of loading (Figure 5), and the benefit of QPLI was demonstrated in its ability to map the kinematics of distinctive populations of fibers (Figures 3, 4). Our ssQPLI system demonstrated high accuracy in fiber orientation and phase retardation, while able to measure complex fiber kinematics in collagenous tissues under continuous dynamic loading situations.

In early QPLI designs, a rotating element(s) required several images to be taken to develop maps of the collagen network (Tower et al., 2002; Lake et al., 2009; Robinson and Tranquillo, 2009; Sander et al., 2011). These systems required 10–20 images over the course of 3–15 s to form the image set, limiting mechanical testing rates to 1.2–4 mm/min, or 30 mm/min with a high speed camera (Quinn and Winkelstein, 2009). In this study, we were able to acquire image sets every 0.02 s and increase the rate to 36 mm/min using standard CCD cameras. While the use of expensive, high speed cameras and fast rotational speeds can be used to achieve similar collection times of 0.04 s (Table 1), the cost of these cameras can be prohibitive, and the system must still take time to serially acquire 10–20 images to generate a single map. This serial acquisition requires short exposure times, which requires increased tissue irradiance or thinner samples to achieve sufficient signal-to-noise ratios. In this experiment, the ssQPLI system was able to capture an image set every 0.02 s with much longer exposure times than previous designs. Reducing the image set acquisition time is important when pairing a QPLI system with mechanical testing, particularly to allow for continuous mechanical loading. In theory, our cameras are capable of taking images every 0.006 s, which can be achieved in future studies that may require imaging of high-speed mechanical loading. If imaging speed is not sufficiently high for some applications, error in orientation maps can be produced due to sample movement during image acquisition, particularly when images are acquired in series. In practice, the rate of tissue displacement must be slow enough that the majority of the tissue at a given pixel location does not move as a complete image set is captured. By definition, snapshot imaging devices, such as our ssQPLI system, can enable mechanical testing at faster loading rates, allowing rate-dependent properties and fiber kinematics in high-speed applications to be studied.

**TABLE 1:** Comparison of QPLI designs. The majority of systems contain a rotating linear polarizer (LP) element in which 10–20 images must be collected in series. The ssQPLI system offers a unique combination of high resolution, efficient light collection, and high acquisition speeds.

Author	Year	QPLI type	Images needed per camera	Image resolution (pixels)	Effective image resolution (mm/px)	Sample thickness (mm)	Mechanical test rate (mm/min)	Time to map (s)
Tower, et al	2002	Rotating LP	20	320 × 240	0.08	0.3–0.5	2–4	3–5
Quinn, et al	2008, 2010, 2011	Rotating LP	20	192 × 396	0.0909	0.43 ± 0.92	30	0.04
Robinson, et al	2009	Rotating LP	20	200 × 200	0.025	—	—	2
Lake, et al	2009, 2010	Rotating LP	15	—	0.025	0.4	1.2	10
Sander, et al	2011	Rotating LP	20	200 × 200	0.025	—	18	—
Wu, et al	2018	Rotating LP	60	616 × 512	0.1	0.5–1.5	75,000	0.006
Woessner, et al	2019	Rotating LP	10	2056 × 2056	0.294	0.03	—	>15
Smith, et al	2019	DoFP	1	500 × 500*	—	1	6–8	0.05
**Blair, et al. (Current)**	**2022**	**DoA**	**1**	**1,200 x 1920**	**0.0275**	**0.5**	**36**	**0.02**

An alternative design to the ssQPLI system using a division of focal plane (DoFP) sensor was previously used to measure collagen orientation in soft tissues (York et al., 2014a; York et al., 2014b; Smith et al., 2019). These DoFP systems also eliminate the rotating polarizer from QPLI systems and create orientation maps with a single image using polarizers at four orientations embedded on the sensor (Table 1). In studies using this DoFP system, images were collected every 0.05 s, which allowed tissue to be tested at 8 mm/min. However, as exposure times decrease with faster frame rates, the collection of a sufficient amount of light becomes more challenging and important. The efficiency of different QPLI systems to convert photons to electrical signal are not well defined, but the camera sensors of the ssQPLI have an efficiency of 76%, compared to 37% of the DoFP sensor. DoFP and rotating LP QPLI designs use linear polarizers, which eliminate half of the collected photons through absorption or reflection. The ssQPLI system uses polarized beamsplitters to sort photons as they enter the collection unit, allowing the cameras to maximize the collection of the incoming photons. Additionally, the extinction ratio ranges from 100:1 to 1,000:1 for the reflected and transmitted outputs of the beamsplitters in the ssQPLI, which exceeds the 58:1 of the DoFP sensor. Perhaps more importantly, the resolution of the ssQPLI system is not reduced by incorporating different polarization angles within a 2 × 2 grid on the sensor. The ssQPLI system can be customized with a wide variety of scientific cameras to meet the temporal or spatial resolution needs of different experiments. Nonetheless, DoFP sensors are less complex than the ssQPLI system and do not require the careful registration of multiple images as with the ssQPLI. As DoFP technology is continually being improved and our ssQPLI system undergoes further refinement, both approaches are viable options for high-speed QPLI.

A challenge in using polarized light to infer collagen orientation and density is that the scattering properties of tissue can randomize the polarization state of the light. Tissues with a thickness of greater than approximately 0.5–1 mm will depolarize the light, and quantitative fiber orientation data cannot be accurately gathered. It is also worth noting that QPLI has no depth resolution and is only capable of detecting the cumulative phase retardation of all fibers through the thickness of the tissue through which light passes. Despite these limitations, the modality is much less expensive than imaging systems that are capable of depth resolution (e.g. optical coherence tomography, multiphoton or confocal microscopy). The ssQPLI system is relatively inexpensive ($6800 USD for essential components) and does not require specialized proprietary components. Furthermore, the acquisition speed of ssQPLI is much faster than those depth resolved imaging systems that require scanning discrete locations in a sample to form an image. High speed acquisition of fiber information over entire tissue samples is critical to understand fiber kinematics during continuous loading and evaluate structure-function relationships in soft tissues. Previous QPLI work has provided information about the fiber kinematics and organization in skin (Woessner et al., 2019), ligaments (Quinn and Winkelstein, 2008, 2009, 2011; Quinn et al., 2010; York et al., 2014a; Smith et al., 2019), tendon (Lake et al., 2009; Lake et al., 2010; Spiesz and Zysset, 2015; Zellers et al., 2021), heart valve tissue (Robinson and Tranquillo, 2009; Tandon et al., 2020), tendon-to-bone interfaces (Wu et al., 2018), and soft tissue analogs and gels (Tower et al., 2002; Lake and Barocas, 2011; Sander et al., 2011; Iannucci et al., 2022). The ssQPLI system increases acquisition speed compared to previous systems without sacrificing resolution or signal-to-noise ratio, which will be particularly helpful for evaluating the time-dependent mechanical properties of viscoelastic tissues, and assessing kinematics during dynamic loading scenarios, such as tendon or ligament injuries.

## Data Availability

The raw data supporting the conclusion of this article will be made available by the authors, without undue reservation.
